# WHO and Gilead Sciences extend collaborative agreement to enhance access to treatment for visceral leishmaniasis

**Published:** 2023-02

**26 JANUARY 2022** - The World Health Organization (WHO) and Gilead Sciences have signed a new agreement for the donation of 304,700 vials of AmBisome (liposomal amphotericin B for injection), for the treatment of visceral leishmaniasis in countries most impacted by the disease, extending their previous agreement to 2025.

The new three-year collaboration (2023-2025), estimated at US$ 11.3 million also includes financial assistance that will support improved coverage and access to diagnosis and treatment for affected communities due to visceral leishmaniasis.

“This longstanding collaboration between WHO and Gilead Sciences exemplifies a successful public-private partnership for advancing the public health agenda and bringing the needed care to affected populations,” said Dr Tereza Kasaeva, Assistant Director-General, a.i., Universal Health Coverage/Communicable & Noncommunicable Diseases. “During the past 9 years, AmBisome, donated by Gilead Sciences, has brought endemic countries, especially in the South-East Asia Region on the verge of eliminating visceral leishmaniasis as a public health problem- a dreaded disease, known for high potential for mortality and outbreak.”

The product donation will benefit affected populations in high-burden countries such as Bangladesh, Ethiopia, India, Kenya, Nepal, Somalia, South Sudan, Sudan, and Uganda along with expansion to include Eritrea, and Yemen as new recipients to treat severe and complicated cases.

Gilead Science’s financial assistance will support WHO’s global efforts to prevent, control, and eliminate visceral leishmaniasis and its impact on the disadvantaged and vulnerable populations in many endemic countries.

“Gilead is proud to renew our commitment supporting the World Health Organization’s efforts to control deaths caused by visceral leishmaniasis in countries most impacted by this life-threatening illness.” said Daniel O’Day, Chairman and Chief Executive Officer, Gilead Sciences. “Through this sustained public-private partnership, we have made significant progress in improving the health outcomes of disadvantaged populations in many endemic countries.”

Visceral leishmaniasis also known as kala-azar, is endemic in 80 countries worldwide. It is the most serious form of leishmaniasis as it is fatal without treatment.

“The World Health Assembly has endorsed the new NTD (Neglected Tropical Diseases) roadmap 2021-2030 and the renewal of this agreement comes at an opportune time as we gear up to achieve ambitious goals set for visceral leishmaniasis and substantially reduce the burden of disease,” said Dr Ibrahima Socé Fall, Director, WHO Department of Control of Neglected Tropical Diseases. “We look forward to this collaboration in our collective efforts towards strengthening services in endemic countries and curbing the menace of this life-threatening condition.”

“The continued donation of AmBisome has improved access to treatment by achieving better outcomes and substantially reducing the duration of treatment from several days to as short as a single injection over few hours, particularly in South-East Asia Region,” said Daniel Argaw Dagne, Unit Head, Prevention Treatment and Care, WHO Department of Control of Neglected Tropical Diseases. “Major countries in elimination settings in South-East Asia Region are at the lowest number of cases and this agreement with Gilead Sciences will enable national programmes to consolidate the gains made over the years and move towards validation of elimination, a crucial step in the pathway of elimination.”

Visceral leishmaniasis is characterized by irregular bouts of fever, weight loss, enlargement of the spleen and liver, and anaemia. The disease affects some of the world’s poorest people and is associated with malnutrition, population displacement, poor housing, a weak immune system, and a lack of financial resources.

The disease is highly endemic in Brazil, the Indian subcontinent, and East Africa.

As a result of this longstanding collaboration, VL morbidity in South East Asia was reduced by more than 82 percent and the case fatality decreased by 95 percent. An estimated 50,000–90,000 new cases occur worldwide each year. Over 90 percent of new cases are reported from seven countries: Brazil, Ethiopia, India, Kenya, Somalia, South Sudan, and Sudan. However, just six years ago, case estimates ranged up to 300,000 cases per year.

Available from: https://www.who.int/news/item/26-01-2023-who-and-gilead-sciences-extend-collaborative-agreement-to-enhance-access-to-treatment-for-visceral-leishmaniasis

